# Atypical Hemolytic Uremic Syndrome Presenting with Diffuse Alveolar Hemorrhage: A Rare Extra-Renal Manifestation

**DOI:** 10.7759/cureus.110039

**Published:** 2026-06-01

**Authors:** Shilpa Mandal, Mekhala Paul, Gourab Bhaduri

**Affiliations:** 1 Nephrology, The Mission Hospital, Durgapur, IND; 2 Critical Care Medicine, The Mission Hospital, Durgapur, IND; 3 Gastroenterology, The Mission Hospital, Durgapur, IND

**Keywords:** adamts13, atypical haemolytic uremic syndrome (ahus), chronic kidney disease (ckd), complement dysregulation, diffuse alveolar haemorrhage (dah), plasma exchange (pex), thrombotic microangiopathy (tma)

## Abstract

We report the case of a 72-year-old male patient with chronic kidney disease (CKD) who presented with diarrhea and subsequently developed thrombotic microangiopathy (TMA) and diffuse alveolar hemorrhage (DAH). Diagnostic workup and clinical course led to a final diagnosis of atypical hemolytic uremic syndrome (aHUS). The patient responded to plasmapheresis and corticosteroid therapy. This case highlights a rare presentation of aHUS with DAH in a patient with pre-existing CKD, and the importance of prompt recognition and multidisciplinary management.

## Introduction

Thrombotic microangiopathies (TMAs) represent a heterogeneous group of life-threatening disorders characterized by the triad of microangiopathic hemolytic anemia, thrombocytopenia, and variable degrees of end-organ injury resulting from widespread endothelial damage and microvascular thrombosis. The clinical spectrum of TMA includes primary entities such as thrombotic thrombocytopenic purpura (TTP) and hemolytic uremic syndrome (HUS), as well as secondary forms associated with infections, autoimmune diseases, malignancy, pregnancy, and drugs. Early recognition and etiological differentiation are critical, as management strategies and outcomes differ substantially among these subtypes [1,2].

Atypical hemolytic uremic syndrome (aHUS) is a rare, complement-mediated TMA resulting from dysregulation of the alternative complement pathway, often due to genetic mutations or acquired autoantibodies affecting complement regulatory proteins such as factor H, factor I, or membrane cofactor protein [3]. This dysregulation leads to uncontrolled complement activation, endothelial injury, and thrombus formation within the microvasculature. Clinically, aHUS predominantly involves the kidneys, frequently progressing to acute kidney injury or chronic kidney disease (CKD), but extra-renal manifestations involving the central nervous system, cardiovascular system, and gastrointestinal tract are increasingly recognized [4].

Pulmonary involvement in TMA, particularly in aHUS, is uncommon and not well characterized. Diffuse alveolar hemorrhage (DAH), defined by bleeding into the alveolar spaces due to disruption of the alveolar-capillary basement membrane, is more typically associated with vasculitides, connective tissue diseases, and certain infections [5]. Its occurrence in the context of aHUS is rare, with only a limited number of cases reported in the literature, and poses a significant diagnostic challenge as it may mimic alternative etiologies such as pulmonary vasculitis or infection. The underlying mechanisms of DAH in aHUS are not fully understood but are hypothesized to involve complement-mediated endothelial injury within the pulmonary microvasculature.

Neurological manifestations in aHUS, including confusion, seizures, and focal deficits, further complicate the clinical picture and may overlap with features of TTP, necessitating careful evaluation including ADAMTS13 activity to guide diagnosis and therapy. The coexistence of pulmonary hemorrhage and neurological involvement in aHUS is particularly rare and underscores the systemic nature of complement-mediated endothelial injury.

We report a case of aHUS in a patient with underlying CKD presenting with prominent neurological symptoms and DAH, highlighting the diagnostic complexity and the need for high clinical suspicion in atypical presentations of TMA.

## Case presentation

A 72-year-old male patient, a known case of CKD for last two years presented with a five-day history of non-bloody, non-mucoid diarrhea. There was no history of fever. The patient’s symptoms improved with supportive care. Stool cultures were negative and stool analysis did not show any ova or parasites.

**Figure 1:**
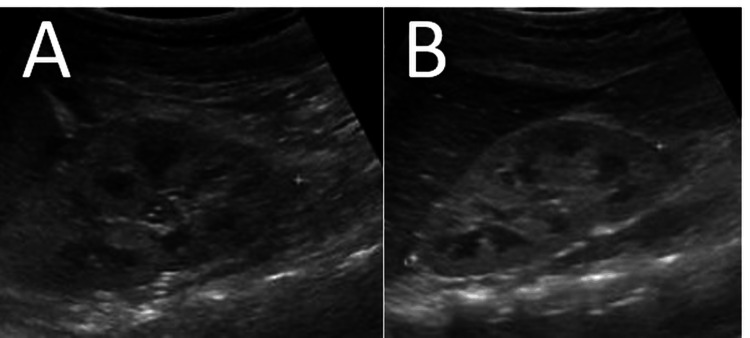
Bilateral shrunken kidneys with increased cortical echogenicity and altered CMD. (A) Left kidney; (B) Right kidney. CMD: Corticomedullary differentiation

Initial laboratory workup revealed elevated urea 165 mg/dL (19.26-42.8 mg/dL) and creatinine 14.1 mg/dL (0.66-1.25 mg/dL). Ultrasonography showed bilaterally shrunken kidneys with increased cortical echogenicity and loss of corticomedullary differentiation (CMD) (Figure 1). Intact parathyroid hormone (iPTH) was elevated - 482 pg/mL (12-65 pg/mL). In view of advanced azotemia, hemodialysis (HD) was initiated.

After two uneventful sessions of HD, the patient developed altered sensorium. Serum urea decreased from 165 mg/dL to 55 mg/dL over two short sessions of HD, and electrolytes remained within normal limits, serum sodium being 137 mEq/L (135-145 mEq/L) and potassium 4.2 mEq/L (3.5-5 mEq/L) , making dialysis disequilibrium syndrome (DDS) unlikely. MRI brain was normal. The patient was intubated in view of worsening sensorium. Cerebrospinal fluid (CSF) analysis was unremarkable.

During the hospital stay, a gradual decline in hemoglobin was noted, though platelet counts remained normal. Initially, lactate dehydrogenase (LDH) was 532 U/L (135-225 U/L), and peripheral smear showed no schistocytes. Repeat evaluation showed LDH of 3,960 U/L and presence of 3% schistocytes. Coagulation parameters (prothrombin time (PT), international normalized ratio (INR), activated partial thromboplastin time (APTT)) were within normal limits. Direct coombs test was negative. The laboratory parameters are listed in Table 1.

**Table 1:** Blood parameters at initiation and follow-up Hb: Hemoglobin; PC: Platelet count; LDH: Lactate dehydrogenase; INR: International normalized ratio; APTT: Activated partial thromboplastin time

	Normal values	August 22, 2025	August 24, 2025	August 26, 2025	August 28, 2025	August 29, 2025	August 31, 2025	September 1, 2025	September 2, 2025	September 3, 2025	September 5, 2025
Hb	13 -17 g/dL	10.7	10.5	8.6	6.6	7.4	6.5	9.8	8.7	8.8	8.7
PC	150-400 x 10^9^ /L	150	160	150	150	150	85	115	50	116	112
LDH	135-155 U/L	-	-	532	3635	-	-	-	-	-	-
Schistocytes	<1%	-	-	<1	3	-	-	-	-	-	-
Urea	19.26-42.8 mg/dL	165	104	52	91	40	43	40	43	77	55
Creatinine	0.66-1.25 mg/dL	14.1	11.3	6.5	3.4	5	5	3.9	3.7	3.8	2
INR	0.8-1.1	0.98	0.96	1.1	1.2	1.19	1.12	1.16	1.13	1.15	1.04
APTT	30-40 seconds	26	26	27	26	28	27	28	30.5	27.4	29.4

A diagnosis of TMA was made. Given central nervous system (CNS) involvement and a PLASMIC (PC, low mean corpuscular volume, active cancer, INR, creatinine) score of 5, TTP was initially considered more likely than HUS. Samples were sent for complement C3 levels and ADAMTS13 activity. Genetic analysis could not be done due to financial constraints.

**Figure 2:**
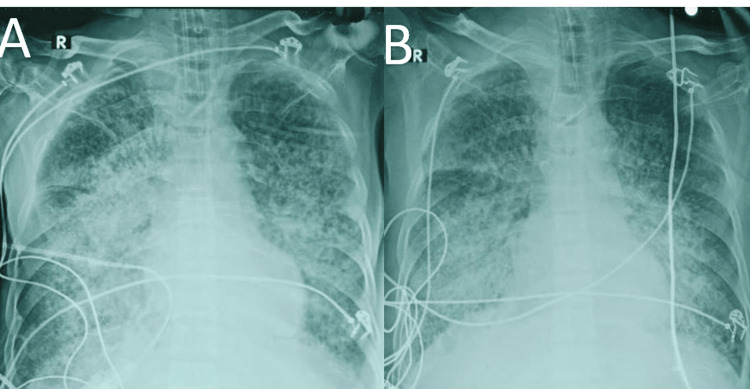
DAH. (A) Before starting PEX and immunosuppression; (B) After two sessions of PEX. DAH: Diffuse alveolar hemorrhage; PEX: Plasma exchange

Plasmapheresis was initiated, and corticosteroids were added due to ongoing hemolysis in the absence of sepsis or disseminated intravascular coagulation (DIC). Subsequently, the patient developed severe hypoxia. Chest X-ray showed diffuse alveolar infiltrates with peripheral sparing (Figure 2A), and blood with clots was noted in the endotracheal tube, suggestive of DAH. CT chest could not be performed as the patient could not be shifted due to severe hypoxia. Bronchoscopy revealed DAH. After few sessions of plasma exchange (PEX), DAH started improving (Figure 2B).

Daily PEX was continued along with fresh frozen plasma (FFP). Methylprednisolone was escalated to 500 mg IV daily for three days, followed by 40 mg IV once daily. Autoimmune workup (antiglomerular basement membrane (anti-GBM), antineutrophil cytoplasmic antibody (ANCA) (myeloperoxidase (MPO), proteinase 3 (PR3)), antinuclear antibody (ANA)) was negative.

**Figure 3:**
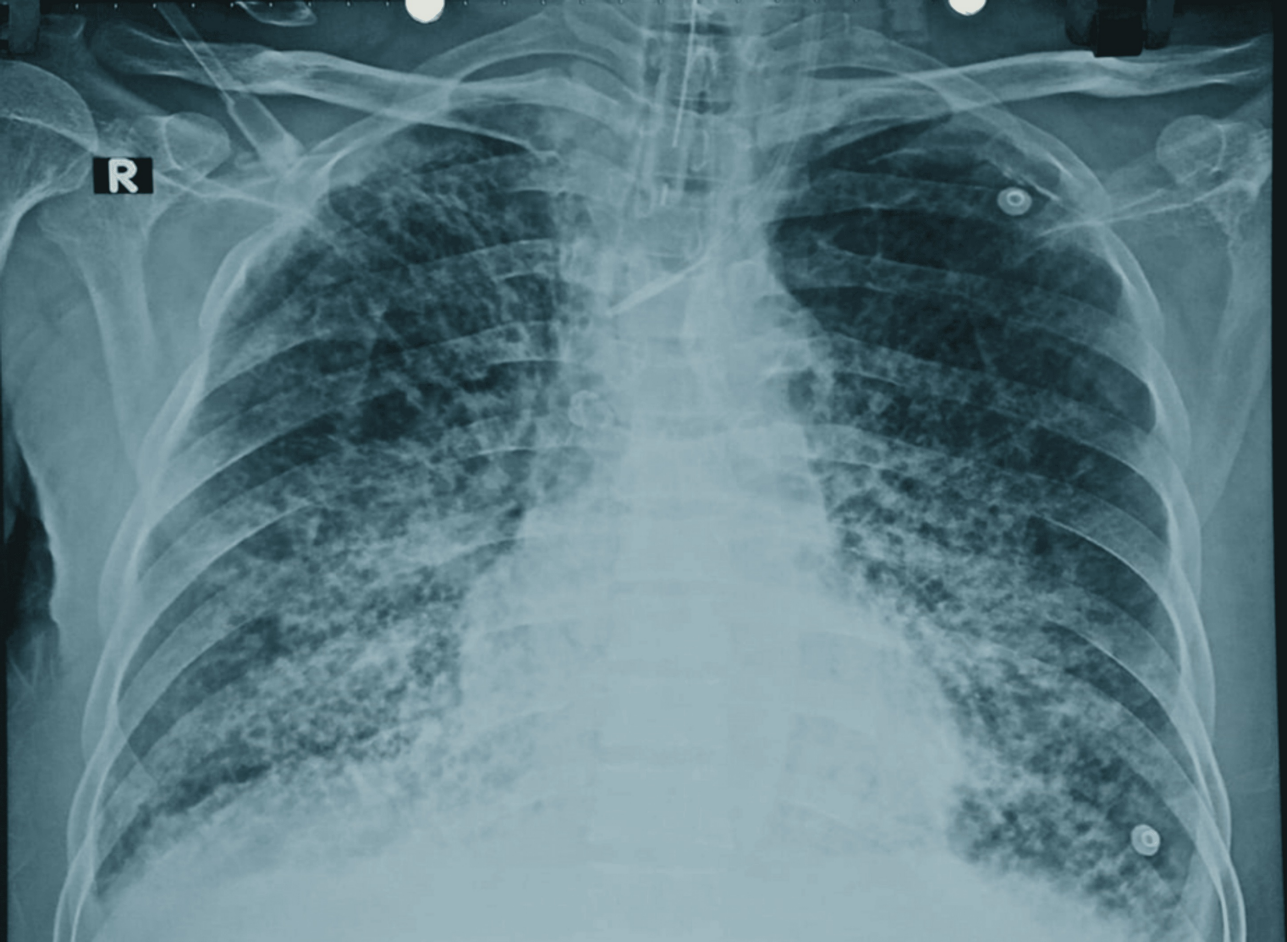
After steroids and five sessions of plasmapheresis

**Figure 4:**
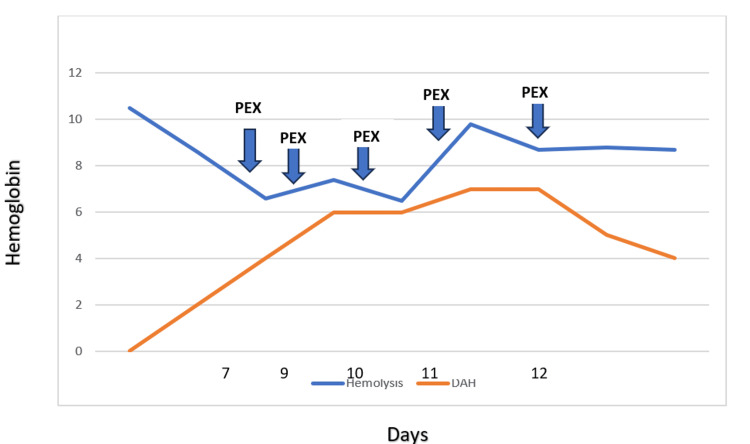
Series of events during hospital stay PEX: Plasmapheresis; DAH: Diffuse alveolar hemorrhage

Test results showed low C3 - 56 mg/dL (90-180 mg/dL) - and ADAMTS13 activity of 22% (ruling out TTP). Thus, a diagnosis of aHUS was made. Following five sessions of PEX and high-dose steroid therapy, hemolysis and DAH improved (Figure 3). The patient regained consciousness and was extubated. Additional PEX sessions were planned due to favorable response. Unfortunately, further therapy was withheld due to financial constraints. The timeline of the entire treatment and response is shown in Figure 4. 

## Discussion

This case highlights the diagnostic complexity of TMA syndromes, particularly in elderly patients with underlying CKD. This case is unique in several ways.

The association of DAH with aHUS is extremely rare. HUS is a form of TMA characterized by hemolytic anemia, thrombocytopenia, and organ dysfunction. It can be triggered by infections, autoimmune diseases, malignancies, pregnancy, or transplantation. aHUS is a rare subtype caused by dysregulation of the alternative complement pathway.

Pulmonary involvement in aHUS, especially in the form of DAH, is exceedingly uncommon and is more typically associated with autoimmune vasculitis or anti-GBM disease.

Ahmad et al. reported a case of an elderly male presenting with generalized weakness, malaise, and hemoptysis [6]. He was found to have hemolytic anemia, thrombocytopenia, and elevated serum creatinine. DAH was confirmed via bronchoscopy. While plasmapheresis yielded only a suboptimal response, initiation of eculizumab led to both clinical and laboratory improvement.

Another case, from Sri Lanka, described a 15-year-old girl who presented with septicaemia and acute kidney injury [7]. On admission, her blood pressure was elevated (180/100 mmHg). She underwent an appendectomy for suspected acute appendicitis. Postoperatively, she developed DAH, respiratory failure requiring mechanical ventilation, and acute renal failure necessitating dialysis. Investigations revealed microangiopathic hemolytic anemia, thrombocytopenia, elevated LDH, reduced complement C3 levels, and a normal coagulation profile. She was diagnosed with aHUS and treated with PEX, corticosteroids, and supportive care, eventually achieving full recovery.

Other reported cases of aHUS presenting with DAH have been associated with infectious triggers such as Epstein-Barr virus (EBV) and H1N1 influenza [8,9].

The patient did not exhibit thrombocytopenia in the early phase. Platelet counts remained stable at 1.5-1.6 x 10^5^/µL thus posing a diagnostic challenge. Serres et al. reported that 44% of patients with histologically confirmed TMA had normal platelet counts, although only a few had no evidence of hemolysis [10].

A case from India described a 20-year-old male patient who developed aHUS following orthopedic surgery [11]. He presented two days postoperatively with oliguria, anasarca, and dyspnea. Labs revealed hemoglobin of 8.5 g/dL, leukocytosis (23 × 10⁹/L), platelet count of 540 × 10⁹/L, serum creatinine of 8 mg/dL (from a baseline of 0.8 mg/dL), serum albumin of 3.4 g/dL, and proteinuria (1.6 g/day) with microscopic hematuria. His hemoglobin dropped further to 4.7 g/dL by day 4 without overt signs of hemolysis. Renal biopsy revealed TMA features; C3 levels were low, and MLPA showed a CFH/CFHR1/CFHR3 gene duplication. Whole-exome sequencing was not pursued due to financial limitations. The patient improved after five sessions of PEX with FFP and corticosteroids.

Our patient was a known case of CKD stage 5, with bilateral shrunken kidneys on ultrasound, making renal biopsy as a part of diagnostic modality unfeasible.

In this case, the presence of TMA features, CNS involvement, low complement C3 levels and negative autoimmune serologies supported the diagnosis of aHUS. While eculizumab is the recommended treatment, cost and availability constraints led to the use of PEX and corticosteroids, which resulted in favorable clinical and laboratory outcomes.

## Conclusions

aHUS must remain a key differential diagnosis in patients presenting with TMA and evidence of end-organ involvement, even when classical features such as thrombocytopenia are absent or only subtly expressed. This underscores the heterogeneity of clinical presentation and the need for a high index of suspicion, particularly in cases with atypical or evolving hematologic parameters. The occurrence of DAH, although an uncommon manifestation, highlights the potential for systemic and life-threatening involvement beyond the renal microvasculature, reinforcing the concept of aHUS as a multisystem disease driven by complement dysregulation.

This case further illustrates the critical importance of a comprehensive and methodical diagnostic approach to TMA, integrating clinical, laboratory, and exclusion-based strategies to differentiate aHUS from other etiologies such as TTP, secondary TMAs, and infection-associated causes. Early recognition is pivotal, as delays in diagnosis and initiation of therapy can significantly worsen renal and overall outcomes. While targeted complement inhibition with therapeutic agents like eculizumab remains the standard of care in many settings, this report also demonstrates that therapeutic PEX and corticosteroids may still offer meaningful clinical benefit, particularly in resource-constrained environments where access to complement inhibitors is limited.
